# Supplementary material for: Protein–protein interaction and non-interaction predictions using gene sequence natural vector
Source: Commun Biol. 2022 Jul 2;5:652. doi: 10.1038/s42003-022-03617-0 (PMC9250521; doi:10.1038/s42003-022-03617-0)
Supplement: Supplementary file 2 — Supplementary Information [file 42003_2022_3617_MOESM2_ESM.pdf]

Supplementary information for the article "Protein-protein interaction and non-interaction predictions using gene sequence natural vector".

Nan Zhao[1], Maji Zhuo[1], Kun Tian[1], Xinqi Gong[*1,2,3]

[1]Institute for Mathematical Sciences, School of Mathematics, Renmin University of China, Beijing, China

[2]Beijing Academy of Artificial Intelligence, Beijing, China

[3]Beijing Advanced Innovation Center for Structural Biology, Tsinghua University, Beijing, China

[*]To whom correspondence should be addressed. Email: xinqigong@ruc.edu.cn

Supplementary Note 1: Five-fold cross-validation results on *H. sapien* and *M. musculus* datasets

The following are the five-fold cross-validation results of seven datasets using support vector machine (SVM) and/or random forest (RF).

1. *H. sapien* and *M. musculus* real datasets (SVM)

Supplementary Table 1. Five-fold cross-validation results on the *H. sapien* and *M. musculus* real dataset using SVM.

Test set	Acc. (%)	Pre. (%)	Sen. (%)	Spe. (%)	MCC (%)	F-score (%)	AUC
H. sapien							
fold 1	75.00	75.90	73.27	76.73	50.03	74.56	0.8254
fold 2	76.98	76.59	77.72	76.24	53.97	77.15	0.8302
fold 3	75.50	73.95	78.71	72.28	51.10	76.26	0.8027
fold 4	80.20	79.90	80.69	79.70	60.40	80.30	0.8370
fold 5	78.96	77.73	81.19	76.73	57.98	79.42	0.8438
Average±SD	77.33±2.22	76.81±2.20	78.32±2.65	76.34±2.65	54.69±4.43	77.54±2.33	0.8278±0.0157
M. musculus							
fold 1	71.05	67.14	82.46	59.65	43.24	74.02	0.8022
fold 2	81.90	76.06	93.10	70.69	65.46	83.72	0.8986
fold 3	77.59	73.53	86.21	68.97	56.01	79.37	0.8499
fold 4	77.59	71.05	93.10	62.07	58.04	80.60	0.8062
fold 5	77.59	74.24	84.48	70.69	55.70	79.03	0.8092
Average±SD	77.14±3.88	72.40±3.45	87.87±4.96	66.41±5.19	55.69±8.00	79.35±3.51	0.8332±0.0413

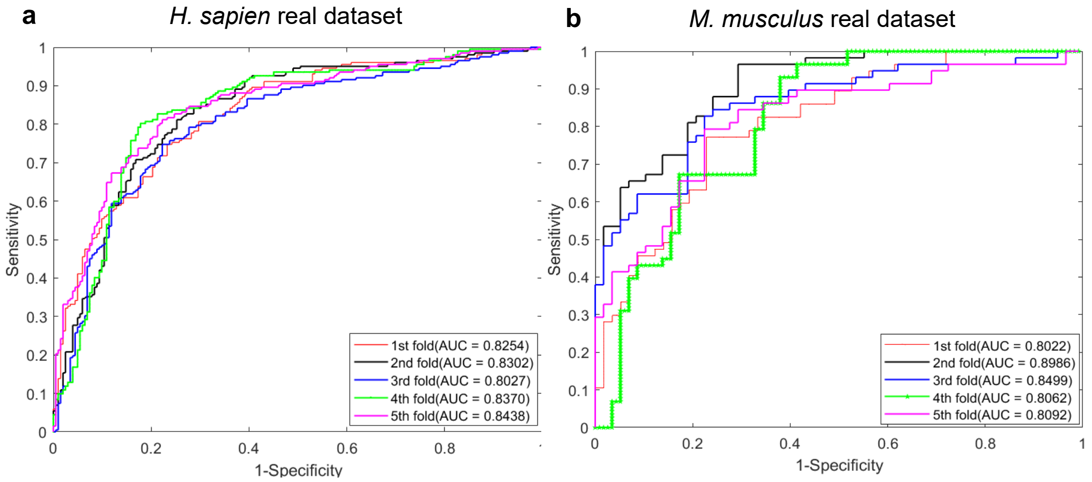

Supplementary Figure 1. The ROC curves for five-fold cross-validation results on the *H. sapien* (**a**) and *M. musculus* (**b**) real dataset using SVM. The numbers in parenthesis indicate the area under curve (AUC).

2. *H. sapien* and *M. musculus* constructed datasets (SVM)

Supplementary Table 2. Five-fold cross-validation results on the *H. sapien* and *M. musculus* constructed datasets using SVM.

Test set	Acc. (%)	Pre. (%)	Sen. (%)	Spe. (%)	MCC (%)	F-score (%)	AUC
H. sapien							
fold 1	96.29	93.09	100.00	92.57	92.83	96.42	0.9818
fold 2	97.28	94.84	100.00	94.55	94.69	97.35	0.9966
fold 3	93.56	88.60	100.00	87.13	87.86	93.95	0.9650
fold 4	94.55	90.54	99.50	89.60	89.55	94.81	0.9765
fold 5	95.30	91.40	100.00	90.59	91.00	95.51	0.9035
Average±SD	95.40±1.45	91.69±2.39	99.90±0.22	90.89±2.84	91.19±2.68	95.61±1.33	0.9647±0.0360
M. musculus							
fold 1	99.12	100.00	98.25	100.00	98.26	99.12	0.9786
fold 2	92.24	92.98	91.38	93.10	84.50	92.17	0.9533
fold 3	93.97	98.11	89.66	98.28	88.26	93.69	0.9834
fold 4	93.97	96.36	91.38	96.55	88.05	93.81	0.9810
fold 5	93.10	98.08	87.93	98.28	86.67	92.73	0.9432
Average±SD	94.83±2.69	96.43±2.64	93.10±3.92	96.55±2.61	89.71±5.31	94.74±2.77	0.9430±0.0184

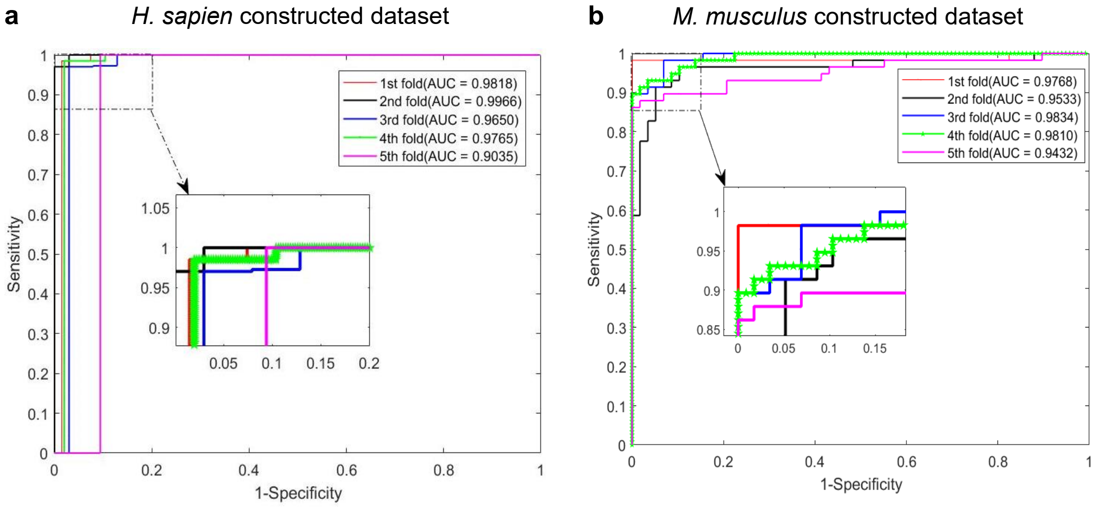

Supplementary Figure 2. The ROC curves for five-fold cross-validation results on the *H. sapien* (**a**) and *M. musculus* (**b**) constructed dataset using SVM. The numbers in parenthesis indicate the area under curve (AUC).

3. *H. sapien* and *M. musculus* real datasets (RF)

Supplementary Table 3. Five-fold cross-validation results on the *H. sapien* and *M. musculus* real dataset using RF.

Test set	Acc. (%)	Pre. (%)	Sen. (%)	Spe. (%)	MCC (%)	F-score (%)	AUC
H. sapien							
fold 1	87.13	88.27	85.64	88.61	74.29	86.93	0.8713
fold 2	84.16	81.08	89.11	79.21	68.65	84.91	0.8416
fold 3	81.19	79.17	84.65	77.72	62.53	81.82	0.8119
fold 4	84.16	84.50	83.66	84.65	68.32	84.08	0.8416
fold 5	85.40	84.86	89.37	84.06	70.99	87.06	0.8540
Average±SD	84.41±2.17	83.58±3.54	86.49±2.61	82.85±4.40	68.96±4.31	84.96±2.18	0.8441±0.0217
M. musculus							
fold 1	83.33	77.14	94.74	71.93	68.47	85.04	0.8333
fold 2	88.79	86.89	91.38	86.21	77.69	89.08	0.8879
fold 3	82.76	78.79	89.66	75.86	66.15	83.87	0.8276
fold 4	80.17	75.36	89.66	70.69	61.46	81.89	0.8017
fold 5	89.66	91.07	87.93	91.38	79.36	89.47	0.8966
Average±SD	84.94±4.10	81.85±6.78	90.67±2.58	79.21±9.14	70.63±7.66	85.87±3.31	0.8494±0.0410

a *H. sapien* real dataset

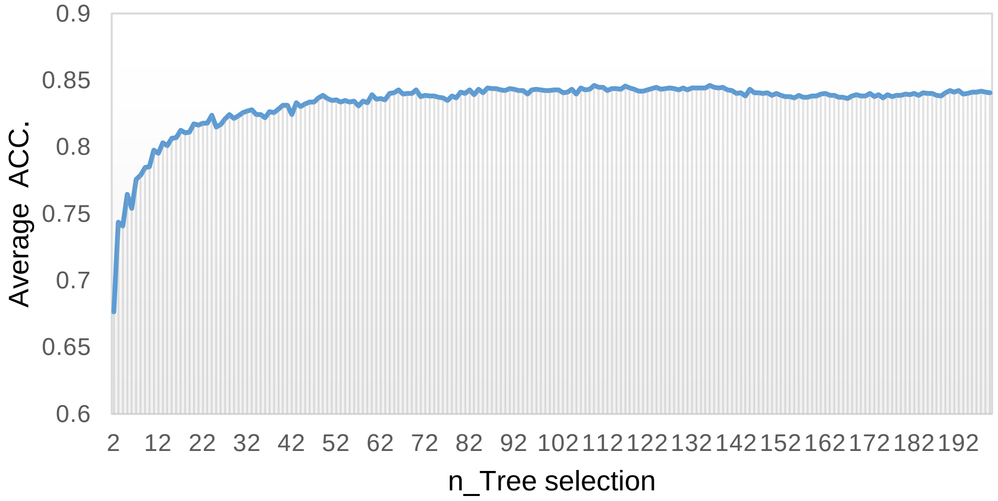

b *M. musculus* real dataset

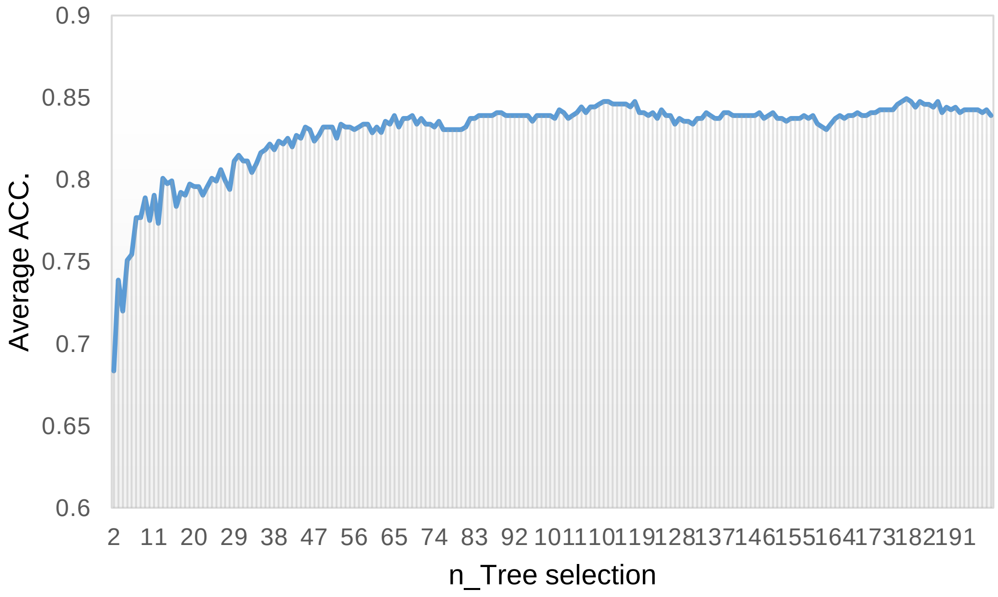

Supplementary Figure 3. Five-fold cross-validation accuracy results on the *H. sapien* (**a**) and *M. musculus* (**b**) real dataset using RF with different n_Tree selections.

4. *H. sapien* and *M. musculus* constructed datasets (RF)

Supplementary Table 4. Five-fold cross-validation results on the *H. sapien* and *M. musculus* constructed datasets using RF.

Test set	Acc. (%)	Pre. (%)	Sen. (%)	Spe. (%)	MCC (%)	F-score (%)	AUC
H. sapien							
fold 1	96.29	93.09	100.00	92.57	92.83	96.42	0.9629
fold 2	97.28	94.84	100.00	94.55	94.69	97.35	0.9728
fold 3	93.56	88.60	100.00	87.13	87.86	93.95	0.9356
fold 4	98.76	98.05	99.50	98.02	97.54	98.77	0.9876
fold 5	95.30	91.40	100.00	90.59	91.00	95.51	0.9530
Average±SD	96.24±1.97	93.19±3.56	99.90±0.22	92.57±4.10	92.78±3.66	96.40±1.82	0.9624±0.0197
M. musculus							
fold 1	97.37	100.00	94.74	100.00	94.87	97.30	0.9737
fold 2	98.28	100.00	96.55	100.00	96.61	98.25	0.9828
fold 3	94.83	100.00	89.66	100.00	90.14	94.55	0.9483
fold 4	96.55	100.00	93.10	100.00	93.33	96.43	0.9655
fold 5	92.24	100.00	84.48	100.00	85.52	91.59	0.9224
Average±SD	95.85±2.38	100.00±0.00	91.71±4.77	100.00±0.00	92.09±4.38	95.62±2.63	0.9585±0.0238

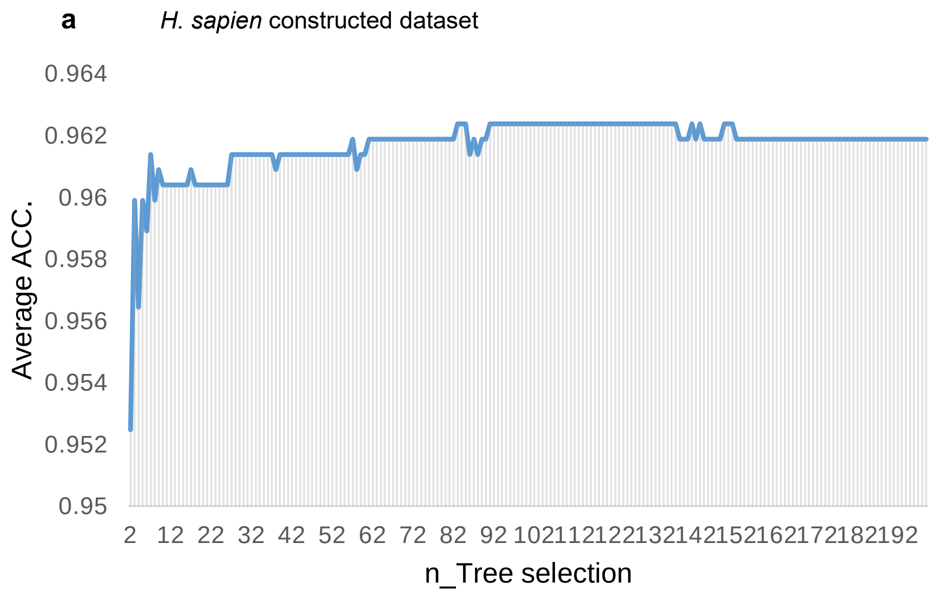

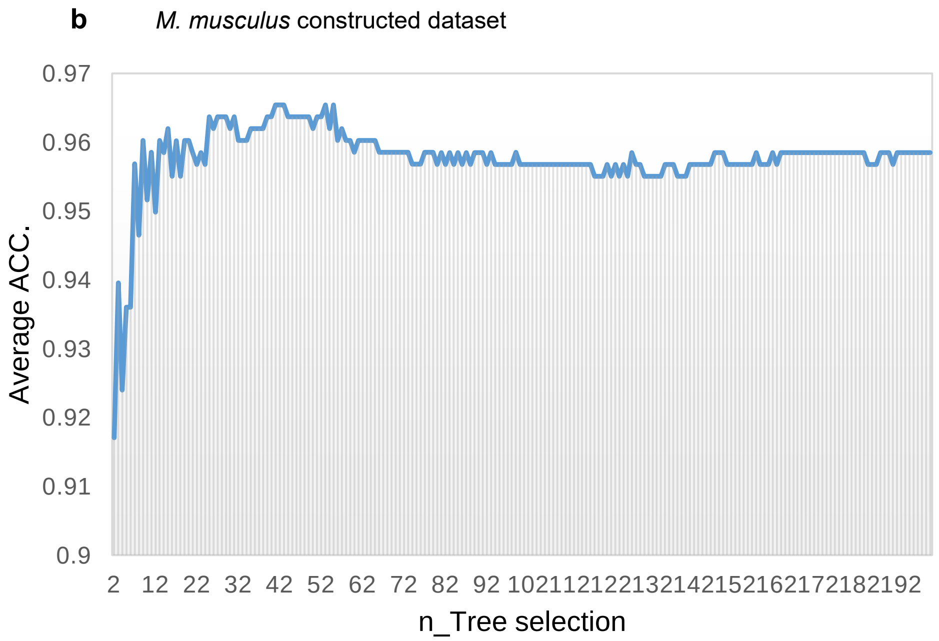

Supplementary Figure 4. Five-fold cross-validation accuracy results on the *H. sapien* (**a**) and *M. musculus* (**b**) constructed dataset using RF with different n_Tree selections.

Supplementary Note 2: Multiple-core network prediction results

Supplementary Table 5. False frequency distributed on each core protein in the prediction of the Q8TBX8-O75175-P31150-Q16828-Q8TAU0-Q9H6S3 pathway.

Proteins	Model 1,m/n	Model 2,m/n	Model 3,m/n	Model 4,m/n
Q8TBX8	1/14	1/14	0/14	0/14
O75175	3/14	2/14	1/14	1/14
P31150	5/14	3/14	0/14	0/14
Q16828	1/14	0/14	0/14	0/14
Q8TAU0	0/13	0/13	0/13	0/13
Q9H6S3	6/13	6/13	5/13	2/13
All	16/82	12/82	6/82	3/82

Note: 0%, 10%, 30% and 40% known interacting protein pairs of the six core proteins were included in the training datasets for constructing models 1, 2, 3 and 4, respectively. m/n represents that there are m false predictions in n PPI related to the core protein in the predicted network.

Supplementary Note 3: Five-fold cross-validation results on *S. cerevisiae*, *D. melanogaster* and *H. pylori* datasets (SVM)

Supplementary Table 6. Five-fold cross-validation results obtained in predicting the *S. cerevisiae*, *D. melanogaster* and *H. pylori* constructed dataset using SVM.

Test set	Acc. (%)	Pre. (%)	Sen. (%)	MCC (%)	F-score (%)	AUC
S. cerevisiae						
fold 1	98.55	99.02	98.07	97.11	98.54	0.9975
fold 2	97.75	98.06	97.42	95.50	97.74	0.9952
fold 3	98.18	99.02	97.32	96.37	98.16	0.9964
fold 4	98.45	99.24	97.64	96.90	98.43	0.9957
fold 5	98.50	99.02	97.96	97.00	98.49	0.9969
Average±SD	98.28±0.33	98.87±0.47	97.68±0.33	96.58±0.67	98.27±0.33	0.9963±0.0009
D. melanogaster						
fold 1	91.29	95.09	87.08	82.88	90.91	0.9489
fold 2	94.66	98.18	91.01	89.57	94.46	0.9737
fold 3	93.82	94.32	93.26	87.65	93.79	0.9754
fold 4	94.41	94.41	94.41	88.83	94.41	0.9665
fold 5	91.90	94.64	88.83	83.96	91.64	0.9633
Average±SD	93.22±1.53	95.33±1.62	90.92±3.03	86.57±2.99	93.04±1.65	0.9656±0.0106
H. Pylori						
fold 1	93.42	99.07	87.65	87.41	93.01	0.9730
fold 2	96.50	98.29	94.65	93.07	96.44	0.9929
fold 3	93.21	94.87	91.36	86.48	93.08	0.9759
fold 4	97.43	98.32	96.50	94.87	97.40	0.9900
fold 5	92.59	97.26	87.65	85.60	92.21	0.9794
Average±SD	94.63±2.18	97.56±1.64	91.56±4.02	89.49±4.19	94.43±2.33	0.9822±0.0088

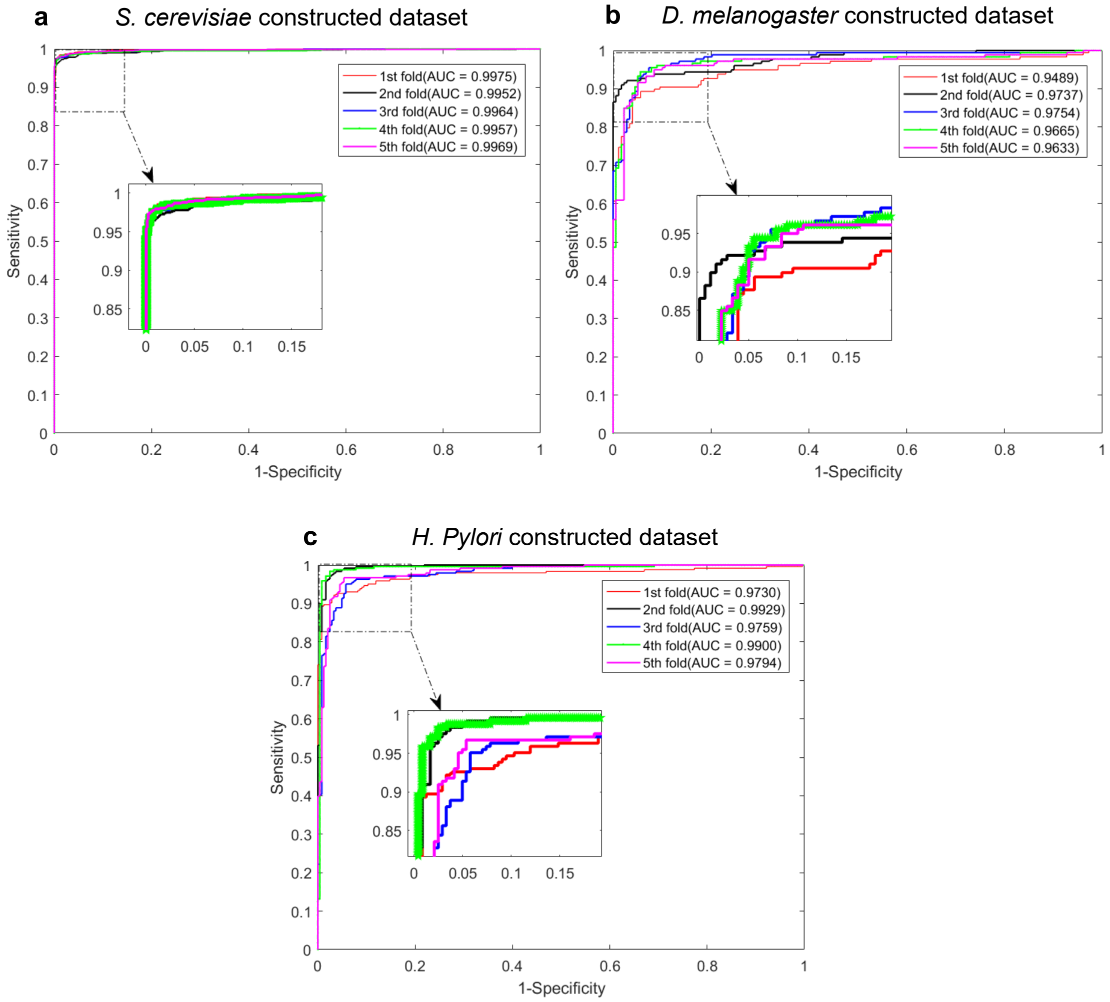

Supplementary Figure 5. The ROC curves for five-fold cross-validation results on the *S. cerevisiae* (**a**), *D. melanogaster* (**b**) and *H. pylori* (**c**) constructed dataset using SVM. The numbers in parenthesis indicate the area under curve (AUC).

Supplementary Note 4: Prediction performance of different feature extraction

Supplementary Table 7. Prediction results based on SVM with NV, NVD and NVDT on *S. cerevisiae*, *D. melanogaster* and *H. pylori* independent test set.

Test species	Method	Acc. (%)	Pre. (%)	Sen. (%)	MCC (%)	F-score (%)
S. cerevisiae	SVM-NV	91.09	88.84	93.99	82.33	91.35
	SVM-NVD	94.90	94.19	95.71	89.82	94.94
	SVM-NVT	99.09	99.04	99.14	98.18	99.09
	SVM-NVDT	99.20	99.35	99.03	98.39	99.19
D. melanogaster	SVM-NV	87.92	91.93	83.15	92.70	76.19
	SVM-NVD	89.04	92.12	85.39	92.70	78.30
	SVM-NVT	93.82	95.88	91.57	96.07	87.73
	SVM-NVDT	94.94	97.62	92.13	97.75	90.03
H. pylori	SVM-NV	90.95	89.96	92.18	89.71	81.92
	SVM-NVD	95.47	96.62	94.24	96.71	90.97
	SVM-NVT	97.94	97.94	97.94	97.94	95.88
	SVM-NVDT	98.77	99.17	98.35	99.18	97.53

Supplementary Note 5: Prediction performance of CCPPI and SVM-NVDT

Supplementary Table 8. Comparison of 10-fold cross-validation results between CCPPI and SVM-NVDT on *S. cerevisiae* training set.

Method	Acc. (%)	Pre. (%)	Sen. (%)	MCC (%)
Codon pair frequency difference (CCPPI)	89.60±0.40	88.10±0.60	91.70±0.10	79.30±0.80
our method (SVM-NVDT)	98.05±0.05	98.14±0.21	97.95±0.19	96.10±0.11

Note: The values in the table are average±standard deviation.

Supplementary Note 6: Datasets

Supplementary Table 9. Datasets used of NVDT.

Dataset Name	Species	No. of interacting protein pairs	No. of non-interacting protein pairs	Length (max, min)
real dataset	*H. sapiens*	1217	1217	(568080, 510)
	M. musculus	347	347	(1125620, 315)
constructed dataset	*S. cerevisiae*	5594	5594	(14733, 168)
	D. melanogaster	1070	1070	(394146, 195)
	H. pylori	1458	1458	(9562, 183)
	H. sapiens	1217	1217	(453160, 510)
	M. musculus	347	347	(1125620, 725)

Note: All positive samples were collected from the DIP database. All negative samples were obtained in two ways. First, the negative samples of real dataset were collected from the Negatome Database, which currently contains experimentally supported non-interacting protein pairs. Second, the negative samples of constructed dataset were pairing proteins at different subcellular locations. Protein subcellular location can be divided into eight categories: cytoplasm, nucleus, cytoplasm & nucleus, mitochondrion, golgi apparatus, endoplasmic reticulum, peroxisome and vacuole. Most importantly, the proteins from the cytoplasmic group and nucleus group can’t pair with proteins from the cytoplasmic & nuclear group.

Supplementary Note 7: Parameters selection

Supplementary Table 10. Parameters selection of each model.

Species	SVM		RF
	**c**	**γ**	**n**
real dataset			
*H. sapien*s	512.0	0.0078125	86
M. musculus	32.0	2.0	180
constructed dataset			
S. cerevisiae	512.0	0.0078125	---
D. melanogaster	128.0	0.5	---
H. pylori	128.0	0.0078125	---
*H. sapien*s	512.0	0.0078125	83
M. musculus	128.0	0.125	90

Note: The dataset where the symbol '---' is located did not use random forest for five-fold cross-validation experiment and independent test set. **c**: the regularization parameter in SVM; **γ**: the Gauss kernel function parameter in SVM; **n**: the number of trees to grow in RF.
