# Supplementary material for: Protein–protein interaction and non-interaction predictions using gene sequence natural vector
Source: Commun Biol. 2022 Jul 2;5:652. doi: 10.1038/s42003-022-03617-0 (PMC9250521; doi:10.1038/s42003-022-03617-0)
Supplement: Supplementary file 3 — Description of Additional Supplementary Files [file 42003_2022_3617_MOESM3_ESM.pdf]

Description of Additional Supplementary Files

File name: Supplementary Data 1

Description: The source data behind the figures in the manuscript.
