# Supplementary material for: Protein–protein interaction and non-interaction predictions using gene sequence natural vector
Source: Commun Biol. 2022 Jul 2;5:652. doi: 10.1038/s42003-022-03617-0 (PMC9250521; doi:10.1038/s42003-022-03617-0)
Supplement: Supplementary file 5 — Reporting Summary [file 42003_2022_3617_MOESM5_ESM.pdf]

nature portfolio

Corresponding author(s): Xinqi Gong

Last updated by author(s): May 15, 2022

Reporting Summary

Nature Portfolio wishes to improve the reproducibility of the work that we publish. This form provides structure for consistency and transparency in reporting. For further information on Nature Portfolio policies, see our Editorial Policies and the Editorial Policy Checklist.

Statistics

For all statistical analyses, confirm that the following items are present in the figure legend, table legend, main text, or Methods section.

n/a	Confirmed	
☐	☒	The exact sample size (n) for each experimental group/condition, given as a discrete number and unit of measurement
☐	☒	A statement on whether measurements were taken from distinct samples or whether the same sample was measured repeatedly
☐	☒	The statistical test(s) used AND whether they are one- or two-sided<br>*Only common tests should be described solely by name; describe more complex techniques in the Methods section.*
☐	☒	A description of all covariates tested
☒	☐	A description of any assumptions or corrections, such as tests of normality and adjustment for multiple comparisons
☒	☐	A full description of the statistical parameters including central tendency (e.g. means) or other basic estimates (e.g. regression coefficient) AND variation (e.g. standard deviation) or associated estimates of uncertainty (e.g. confidence intervals)
☒	☐	For null hypothesis testing, the test statistic (e.g. F, t, r) with confidence intervals, effect sizes, degrees of freedom and P value noted<br>*Give P values as exact values whenever suitable.*
☒	☐	For Bayesian analysis, information on the choice of priors and Markov chain Monte Carlo settings
☒	☐	For hierarchical and complex designs, identification of the appropriate level for tests and full reporting of outcomes
☒	☐	Estimates of effect sizes (e.g. Cohen's d, Pearson's r), indicating how they were calculated

Our web collection on statistics for biologists contains articles on many of the points above.

Software and code

Policy information about availability of computer code

Data collection	The positive datasets consisted of interacting protein pairs collected from the public Database of Interacting Proteins (DIPs: https://dip.doe-mbi.ucla.edu/dip/). The negative dataset was composed of non-interacting protein pairs obtained in two ways. First, protein pairs in different subcellular compartments were obtained, based on the assumption that proteins within different subcellular localizations tend not to interact. The other negative samples were derived from the Negatome database version 2.0 (http://mips.helmholtz-muenchen.de/proj/ppi/negatome).
Data analysis	Data were classified and selected according to the species of proteins. No software was used for data clustering and other analysis.

For manuscripts utilizing custom algorithms or software that are central to the research but not yet described in published literature, software must be made available to editors and reviewers. We strongly encourage code deposition in a community repository (e.g. GitHub). See the Nature Portfolio guidelines for submitting code & software for further information.

Data

Policy information about availability of data

All manuscripts must include a data availability statement. This statement should provide the following information, where applicable:

- Accession codes, unique identifiers, or web links for publicly available datasets
- A description of any restrictions on data availability
- For clinical datasets or third party data, please ensure that the statement adheres to our policy

The data used in this work are available at https://github.com/Zhaonan99/NVDT.git and Supplementary Data 1. Supplementary Data 1 is a .xlsx file that includes data for reproducing Figure 1. Supplementary Table 7 includes data for reproducing Figure 2. The interacting protein pairs discussed have been deposited in the

DIPs database, the real non-interacting protein pairs are accessible through the Negatome Database 2.0, and the gene sequence of each protein is available from NCBI database. Other information is available from the corresponding author on reasonable request.

Field-specific reporting

Please select the one below that is the best fit for your research. If you are not sure, read the appropriate sections before making your selection.

☒ Life sciences ☐ Behavioural & social sciences ☐ Ecological, evolutionary & environmental sciences

For a reference copy of the document with all sections, see nature.com/documents/nr-reporting-summary-flat.pdf

Life sciences study design

All studies must disclose on these points even when the disclosure is negative.

Sample size	Seven datasets were collected in this way, including 11,188 protein pairs for S. cerevisiae, 2,140 protein pairs for D. melanogaster, and 2,916 protein pairs for H. pylori, 2434 protein pairs for H. sapiens, and 694 protein pairs for M. musculu, with interacting pairs and non-interacting pairs each accounting for half. Among them, due to different negative sample collection methods, H. sapiens and M. musculu have two types of data sets respectively.
Data exclusions	Samples with fewer than 50 amino acids and >40% pairwise sequence identity to one another were excluded.
Replication	The training parameters are determined by five-fold cross-validation, so as to train the model and verify the test set. After the test, the repeatability can be guaranteed through the determined training parameters.
Randomization	We randomly assign positive and negative samples to form training set and test set. When negative samples were constructed according to different subcellular locations, negative samples were randomly selected as the number of positive samples.
Blinding	Positive samples were downloaded from the interaction database, with some protein pairs removed according to the above conditions. There are two types of negative samples. One is made by pairing proteins at different subcellular locations, and the protein pairs present in the positive samples are eliminated, and the selection of negative samples was random without any cluster analysis. The other was downloaded from the Negatome database, where pairs of proteins are selected according to the species being studied.

Reporting for specific materials, systems and methods

We require information from authors about some types of materials, experimental systems and methods used in many studies. Here, indicate whether each material, system or method listed is relevant to your study. If you are not sure if a list item applies to your research, read the appropriate section before selecting a response.

Materials & experimental systems

n/a	Involved in the study	
☒	☐	Antibodies
☒	☐	Eukaryotic cell lines
☒	☐	Palaeontology and archaeology
☒	☐	Animals and other organisms
☒	☐	Human research participants
☒	☐	Clinical data
☒	☐	Dual use research of concern

Methods

n/a	Involved in the study	
☒	☐	ChIP-seq
☒	☐	Flow cytometry
☒	☐	MRI-based neuroimaging
